# The Seity Check-In: A novel tool for assessing momentary well-being

**DOI:** 10.1371/journal.pmen.0000429

**Published:** 2025-09-23

**Authors:** Jamie McCreary, Harold Stanislaw, Katrina Hawley, Lia Romeo

**Affiliations:** 1 Department of Psychology, California State University, Stanislaus, Turlock, California, United States of America; 2 Departments of Psychology and Music, California State University, Long Beach, California, United States of America; 3 Department of Kinesiology and Public Health, California Polytechnic State University, San Luis Obispo, California, United States of America; Santa Casa de Sao Paulo School of Medical Sciences, BRAZIL

## Abstract

The Seity Check-In (SCI) is a new, multidimensional measure of momentary well-being that features both hedonic and eudaimonic elements. It imposes minimal cognitive demands on respondents, asking them to report their current levels of energy, direction, belonging, and joy by selecting one of five “happy face” emojis for each dimension. This task can be completed in just a few seconds. The psychometric model underlying the SCI is formative, with each of the four dimensions contributing directly to well-being. A preregistered data analysis of 564 individuals who were living in the United States and completed the SCI and other instruments found high correlations (*r* = .84 to.86) with two established measures of well-being, providing strong evidence for criterion validity. Furthermore, responses for each SCI dimension were highly correlated (*r* = .80 to.85) with items adapted from other scales designed to assess the same dimension, providing strong evidence for construct validity. Analyses of open-ended mood descriptions corroborated the psychometric findings. We conclude that the SCI offers considerable utility for assessing well-being, especially in applied situations.

## Introduction

Do mental health professionals really need yet another instrument to assess well-being? A staggering number of options already exist; a recent review that considered only one of several different classes of well-being assessments identified 99 measures [1]. However, despite the plethora of available choices, we argue here that another instrument is, in fact, needed. This need stems from important methodological gaps in the assessments that are currently available. We describe these gaps below and present psychometric evidence for the validity and utility of a novel instrument that bridges them: the Seity Check-In (SCI).

Our treatment here is based largely on practical considerations. Researchers may assess well-being for purely theoretical reasons, hoping to better understand it for the same reasons they may study intelligence or motivation as psychological constructs. However, well-being can also be examined as an outcome. This orientation is consistent with Martin Seligman’s charge – in his 1998 president’s address to the American Psychological Association – that psychology’s “larger mission” is “making the lives of all people better” [2]. From this perspective, well-being is assessed to determine the factors that impact well-being, and whether interventions or policies designed to improve it have their intended impact. This practical orientation underlies the arguments we describe below.

One gap filled by the SCI is best understood by considering the general approaches used to assess well-being. Economists, policy makers, and others often examine purely objective measures that they believe correlate with or determine well-being, such as income, health, employment opportunities, or access to food, housing, and other critical resources. However, psychologists generally prefer more subjective measures that are typically assessed through surveys (see [3] and [4] for comprehensive reviews). The earliest surveys asked respondents to indicate if they felt happy or were satisfied with their lives; these assess hedonic well-being (HWB). One HWB measure, known familiarly as Cantril’s Ladder [5], is used in the World Happiness Report [6] and incorporates a visual model, with the top and bottom of the ladder representing the “best possible life” and “worst possible life,” respectively.

Another popular HWB measure is the Satisfaction with Life Scale (SWLS [7]). The SWLS captures perceptions of one’s life with five items, including “In most ways, my life is close to my ideal,” and “I am satisfied with my life.” A 7-point agreement scale is used to make responses. The SWLS does not tap perceptions of specific life domains such as finance or health; it focuses instead on perceptions of global well-being.

This contrasts with the Positive and Negative Affect Schedule (PANAS [8]), which also assesses HWB but focuses on emotions rather than perceptions. The PANAS presents 10 positive and 10 negative adjectives that could potentially describe one’s mood or internal state. Respondents use a 5-point scale to indicate how well each adjective applies to themselves. A short form of the PANAS, with five positive and five negative adjectives, is also available [9].

More recently – and especially following the growing interest in positive psychology – well-being surveys have examined psychological functioning, such as having a sense of purpose. These instruments assess eudaimonic well-being (EWB). A widely used EWB measure is the PERMA-Profiler [10], which assesses Seligman’s [11] five pillars of well-being – positive emotions, engagement, relationships, meaning in life, and accomplishment – as independent dimensions. The PERMA-Profiler presents 15 items (plus eight filler items), including “How often do you feel joyful?” and “To what extent do you feel excited and interested in things?” An 11-point response scale is used, with anchors that vary depending upon the item.

The Flourishing Scale (FS [12]) is also frequently used to assess EWB. It presents eight indicators that individuals respond to using a 7-point agreement scale. These indicators capture the respondent’s self-perceived success in areas such as relationships, self-esteem, purpose, and optimism.

The instruments described above are just a small sampling of the options that are available to assess well-being. Meiselman identified 20 well-being instruments [13], Dronavalli and Thompson found 27 [14], Cooke et al. located 42 [15], and Linton et al. reviewed 99 [1]. Clearly, there is considerable disagreement about how to understand and measure well-being: Cooke et al. found the variability across measures “striking” and urged researchers and practitioners to pay close attention to what is being assessed when choosing instruments. Little progress has been made to resolve these issues since the comprehensive reviews were published a decade ago. Only a handful of new well-being measures have been proposed in the interim, with most designed for use in specific applications. For example, the EQ Health and Wellbeing tool was created to evaluate the outcomes of health and social care interventions [16], while the Pitt Wellness Scale was developed using a crowd-sourced approach to assess well-being in university populations [17].

The gap that emerges from all of these disparate views of well-being is that many current instruments tend to assess only HWB or EWB. For example, the PANAS does not ask about direction or purpose, and the FS does not ask about happiness. This is problematic because, if the goal of assessing well-being is to identify potentially beneficial interventions, practitioners must know whether to focus their efforts on (say) helping individuals experience joy, or helping them find direction in their lives. Well-being is obviously compromised when individuals who have purpose in their lives also feel miserable, but detecting this requires a hybrid instrument that includes both hedonic and eudaimonic elements.

One option is the Mental Health Continuum-Short Form (MCS-SF [18]), which assesses three areas of well-being: emotional, psychological, and social. Users evaluate 14 items with a 6-point response scale to indicate how often they have experienced feelings such as “calm and relaxed” or “active and vigorous” over the past month. The Warwick-Edinburgh Mental Well-Being Scale (WEMWBS [19]) also assesses both HWB and EWB. Fourteen questions ask about the frequency of positive perceptions of the self, including both feeling good (“I’ve been feeling cheerful”) and functioning well (“I’ve been interested in new things”). The Short-WEMWBS (SWEMWBS [19]) is a briefer, 7-item version that is also widely used; it emphasizes EWB more than HWB.

The overwhelming variety in well-being measures points to a second gap. There is broad agreement that well-being is composed of at least two dimensions (HWB and EWB), and some psychologists (e.g., [3]) have argued for far more dimensions. Despite this, most instruments generate a single, overall well-being score instead of a set of scores that provides insights regarding the different elements that contribute to well-being. There are a few notable exceptions: The MCS-SF generates three scores, the PERMA-Profiler generates five, and the Well-Being Profiler (WB-Pro [3]) generates as many as 15. However, if the goal of well-being assessment is to yield actionable data, the majority of instruments fail to deliver. An instrument that simply identifies individuals as experiencing high or low levels of well-being without providing more detailed, diagnostic information does not guide practitioners toward specific interventions for individuals who could benefit from help.

Multidimensional instruments can address this concern, but a gap found in most of these instruments stems from the large number of items they usually present to respondents. For example, there are 14 items in the MCS-SF, 23 in the PERMA-Profiler, and 48 in the long form of the WB-Pro. Respondents should be able to cope with this many items when they complete the instruments once or at widely spaced intervals; however, if they are asked to complete the instruments every day, boredom, response fatigue, and other issues could easily reduce the validity of their responses. Few instruments are so brief that they can be repeatedly administered at short intervals, as would be necessary in longitudinal studies of well-being at fine-grained temporal intervals, or in pre/post evaluations of intervention impacts.

Another gap emerges from noting that well-being can be considered both a relatively stable *trait*, and a *state* that fluctuates in response to lived experiences [4,20]. Some trait measures, such as the WB-Pro, simply ask respondents about their well-being, without referencing a particular time period to consider in formulating an answer. Others specify a time span to consider when responding. For example, the SWEMWBS asks respondents about their well-being over the previous 2 weeks, while the PERMA-Profiler indicates – on some items, but not others – to consider experiences during the preceding week. The underlying assumption is that respondents will answer based on their “average” experience, ignoring fluctuations in well-being that may have occurred around this average. We question this assumption below, but note here that it is based upon a trait view of well-being. This explains why test-retest data is presented as evidence of the instruments’ psychometric soundness. Test-retest reliability is meaningful only if there is a strong expectation that well-being will be at similar levels when the instrument is repeatedly administered, which is a trait view of well-being.

Understanding well-being as a trait is important for some situations, but understanding well-being as a state is also important. Feelings of well-being rise and fall across time [21,22], so examining well-being as it unfolds in daily life is “imperative” [23]. Furthermore, practitioners delivering an intervention assume (implicitly or explicitly) that well-being is a state, and therefore capable of being changed by the intervention. In this context, a trait measure of well-being is ill-suited to determining the intervention’s impact: Instruments that ask respondents how they felt over the past few weeks are clearly incapable of detecting changes in well-being following the administration of an intervention – if respondents actually adhere to the time frame instructed by the instruments.

This latter point, regarding adherence to instructions, illustrates another gap in existing methods for assessing well-being. Accurately recalling how one felt days or weeks ago is a difficult task, rendered even more demanding by combining all of the feelings experienced during that interval into a single, representative response for each item in an instrument. Summating experiences in this manner can be influenced by a variety of factors, including heuristics that favor some feelings over others, and recall biases such as recency [24–26]. In short, many instruments place unreasonable cognitive demands upon respondents.

The cognitive challenges and biases associated with recalling and aggregating feelings over extended time periods are avoided by *momentary* assessments of well-being. These ask about well-being “now” or “in the moment” [26]. Momentary assessments are explicitly state measures. Moreover, since momentary assessments can be administered repeatedly and at short intervals, they are ideally suited for longitudinal tracking of well-being and evaluating the immediate impact of well-being interventions.

Ecological momentary assessments, which are completed in natural environments, can potentially yield even more valid measures of well-being because they measure it in situ [27,28]. Prompts delivered to a smartwatch or smartphone can ask users to report their current emotional states in real time, while they are at work or school, in the gym, or spending time with friends [21,29]. These methods are especially valuable for practitioners since they reveal the ebb and flow of feelings that impact functioning and clinical prognosis [27]. Kim et al. predict a future in which the “temporally dense” data provided by ecological momentary assessments enable creation of “an evidence-based, safe, and effective health-care system” that delivers improved physical and mental well-being [30].

However, even in natural settings, care is needed to ensure that well-being assessments do not induce demand characteristics or other response biases. This concern is illustrated by the mPERMA, which is a momentary adaption of the PERMA-Profiler [23]. Respondents rate 15 items, so fatigue or boredom can set in towards the end of the assessment. Disengagement seems especially likely if the mPERMA is completed day after day – or multiple times in a single day – which is common for momentary assessments. Furthermore, the mPERMA uses a sliding response scale that ranges from 0 (*not at all*) to 100 (*extremely*). In theory this response scale offers considerable mathematical precision, but do respondents really need to decide whether they are at a 73 or 78 on any given item – and how much does this deliberation detract from their current feelings?

The ideal momentary well-being assessment should be so brief and so simple that respondents do not regard its completion as imposing a burden. This mandates presenting only a few items, coupled with response options that are optimized for cognitive efficiency rather than mathematical precision; users should not need to question how their feelings map onto the instrument’s response options. Otherwise, once users begin to respond they may shift their attention away from how they felt immediately before they were prompted to respond and focus instead on how they feel “in the moment” – that moment being “I’m completing a well-being instrument now (again).”

A final gap in methodology relates to the psychometric evidence used to support the vast majority of well-being instruments. As we noted above, well-being assessments should yield actionable data. Ideally, practitioners should be able to tailor interventions based on client responses. For example, a client who completes the FS and has a low score for “my social relationships are supportive and rewarding” but a high score for “I lead a purposeful and meaningful life” seems more likely to benefit from an intervention designed to build relationships than one designed to generate feelings of purpose. However, this usage is inconsistent with the psychometric evidence that supports the FS, which – like the evidence for many other well-being measures – is based upon a reflective measurement model.

**Fig 1:**
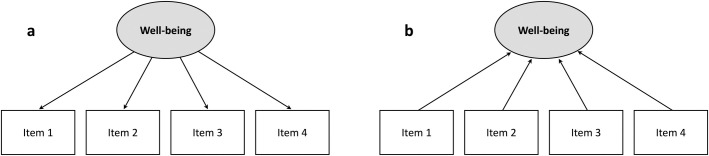
Reflective (a) and formative (b) psychometric measurement models.

In reflective models, item responses are considered *indicators* of well-being rather than *contributors* to well-being (see [3] and [31] for more on this distinction). Thus, targeted interventions based on responses to particular items are not guaranteed to impact well-being, because the direction of causality – according to the psychometric model – flows in the wrong direction for this to occur (Fig 1a). Furthermore, reflective models lead to the improbable prediction that any improvement in the respondent’s sense of belonging or purpose should be accompanied by improvements in all the other elements of well-being.

By contrast, a formative model explicitly states that the assessed dimensions combine to generate the well-being experienced by the respondent (Fig 1b). Thus, if the responses on a formative instrument suggest a problem with relationships but not with feelings of purpose, interventions that succeed in improving relationships should improve well-being, while interventions that target feelings of purpose should not; each element contributes separately to well-being.

Reflective and formative models also differ in the importance they place upon the individual items that comprise the instrument. The items in reflective models are interchangeable; adding or dropping one typically has little impact on the instrument’s overall validity. This property enabled Stewart-Brown et al. to start with the 14-item WEMWBS, and then delete seven items (following a Rasch analysis) to derive the SWEMWBS without compromising the instrument’s psychometric properties [19]. By contrast, every item in a formative model is essential; a low score on one cannot be compensated for by a high score on another.

To summarize, significant gaps exist in current methods for assessing well-being. The ideal instrument for practitioners yields multidimensional scores derived from both hedonic and eudaimonic items. It should provide a momentary measure of well-being and impose no significant cognitive demands upon the respondent. The instrument should be capable of repeated administration at short intervals. Furthermore, it should be supported by psychometric evidence that is based upon a formative measurement model. Several instruments bridge one or more of these gaps, but the SCI appears to be the only measure to date that bridges all of them.

### The Seity Check-In

The SCI was created by Seity Health, LLC [32] and is brief, easy to complete, and suitable for frequent use. The instrument originated from discussions between Seity family medicine physicians and their patients, which led to the creation of 61 proprietary, whole-person health measures designed to assess well-being attributes beyond the physical attributes usually tracked by physicians. An exploratory factor analysis of the responses to these whole-person health measures was conducted using Seity archival data. The results suggested the measures were related to four dimensions that align with the well-being literature. The SCI was created to assess these four dimensions with a single item for each: Respondents indicate their energy, their sense of direction or purpose, their feeling of belonging with others, and their experience of joy. Direction affects EWB and joy impacts HWB, while energy and belonging contribute to both EWB and HWB. Thus, the SCI measures both EWB and HWB and is more holistic than assessments that focus largely or exclusively on one or the other type of well-being. Respondents indicate how they feel at the time of completion, so the assessment is momentary and can be administered daily – or even several times per day.

Early versions of the SCI used sliding response scales, with scores for each of the four dimensions ranging from a low of 0 to a high of 100. However, user feedback suggested this response methodology was awkward and unnecessarily precise, reducing engagement with the instrument. Accordingly, the current version of the SCI uses a 5-point response scale with smiley-face emojis attached to each response option. Scores on each dimension (energy, direction, belonging, and joy) can range from 1 (for the emoji with the largest frown) to 5 (for the emoji with the happiest grin). Humans are exceptionally skilled at interpreting facial expressions [33] and emojis are ubiquitous in many societies [34], so the smiley-face response scale imposes minimal cognitive demands. Once respondents have used the SCI often enough to become familiar with the four dimensions it assesses, the tool can be completed in just a few seconds, eliminating concerns with boredom or response fatigue.

The SCI is posted at https://osf.io/75ym3/?view_only=bd203c139d714d6092d013372082ca31. The four scores it yields can be averaged or summed to provide an overall well-being score, but practitioners can consider each of the four SCI dimensions separately for diagnostic applications. The underlying psychometric model is formative: The four components of the SCI are considered determinants of (or contributors to) well-being. This implies that practitioners can use the SCI to identify which dimension(s) should be targeted to improve well-being and which are at satisfactory levels.

### Present study

As noted above, the SCI has considerable potential – especially for practical applications. Research incorporating the SCI is emerging (e.g., [35]), but formal evaluations of the tool’s psychometric properties have not been conducted. This study provides the first psychometric evidence for the validity of the SCI, using a preregistered analysis.

The SCI’s criterion validity was assessed by determining how SCI scores correlate with SWEMWBS and FS scores. Further evidence for criterion validity was obtained by examining how SCI scores correlate with the valence of each respondent’s description of their mood.

The SCI’s construct validity was assessed by examining how responses to each SCI dimension correlate with items from other instruments that measure those same dimensions. Thus, SCI energy responses were correlated with responses to items adapted from the Subjective Vitality Scale (SVS [36]). SCI direction responses were correlated with responses to items adapted from the Purpose in Life Questionnaire (PLQ [37]). SCI belonging responses were correlated with responses to items adapted from the General Belongingness Scale (GBS [38]). Last, SCI joy responses were correlated with responses to items adapted from the State Joy Scale (SJS [39]). Additional evidence regarding construct validity was obtained by examining how well the ratings for each SCI dimension aligned with self-reported mood descriptions.

Psychometric studies often examine reliability as well as validity. However, internal consistency reliability is meaningless for formative instruments [31], so it was not examined for the SCI. Similarly, test-retest reliability was not examined because the SCI assesses state well-being; there is no expectation of a strong correlation between responses across multiple administrations.

## Materials and methods

### Analysis preregistration

This study utilized a preregistered data analysis that was posted on the Open Science Framework (OSF) after data collection had been completed (see data availability statement). The analysis had not begun when the posting was made, but some of the criteria for excluding respondents had already been applied because they relate to paying study participants. The following sections summarize the major features of this study’s methodology; additional details can be found in the OSF post.

### Participants

The protocol for this study was approved by the California State University, Stanislaus Psychology Department Institutional Review Board (Protocol #21–94). Respondents were recruited through Amazon’s Mechanical Turk (MTurk). They were required to be at least 18 years old and living in the United States at the time of survey completion. A total of 846 respondents began the survey but 282 were excluded – most often for failing an attention check (Table 1). The remaining 564 respondents ranged in age from 21 to 80 years (*M* = 40.87, *SD* = 11.49); 55.1% were male, 44.0% were female, 0.7% were non-binary, and 0.2% chose not to disclose their gender. Most respondents had some postsecondary education: 17.0% held a 2-year degree or certificate, 45.0% held a 4-year degree, and 17.9% held a graduate degree.

**Table 1:** Number of respondents excluded for each reason.

Reason for exclusion	Number excluded
Abandoned study before completing survey	33
Not living in the United States	3
Postal code differed from state of residence	19
Failed one or both attention checks	184
Did not answer all well-being items	10
Sourced the mood response from the Internet	7
Duplicate respondent	24
Completed survey but did not request MTurk payment	2

### Materials

Participants completed an online survey administered through Qualtrics. The survey began with a consent form, followed by demographic questions. Two blocks of items were presented next. One displayed the four SCI items on a single page. The other block presented 39 items across five pages (eight items per page): two attention checks, the SWEMWBS (seven items), the FS (eight items), six items from the SVS, four items from the PLQ, six items from the GBS, and six items from the SJS. The two blocks were presented in random order, with items in the longer block also presented in random order. Last, an open-ended question asked respondents to describe their mood in 25 words or less (although the word limit was not enforced). The final survey page was a debriefing form.

The SCI used a 5-point “happy face” response scale, while the other items used a 7-point Likert scale. These latter items were rephrased from their original form to include the word “today,” ensuring their suitability for momentary assessment. Other researchers used a similar approach to adapt the PANAS [8] and the SWEMWBS [40] for momentary assessment. The full text of the survey is available in the supplemental materials, at https://osf.io/geptf/?view_only=a4a192d940d642278d38cea5fd928132.

### Procedure

The survey was posted daily on MTurk from December 15–23, 2021. Respondents indicated their consent by clicking on an “I Agree–Begin Survey” button at the bottom of the consent form. They were compensated US $1.00 for completing the survey, unless they had already attempted the survey, or their response to the mood question was copied from an Internet source (suggesting completion by a bot), or they failed an attention check. To pass these checks, respondents were required to select *strongly disagree* for “Today I am 7 years old,” and *strongly agree* for “Today I am completing a survey.”

### Scoring

Each SCI response was assigned a value ranging from 1 (for the saddest emoji) to 5 (for the happiest emoji). The total SCI score was then found by adding the energy, direction, belonging, and joy scores. Unit scoring the SCI in this manner yielded a possible total score ranging from 4 to 20. We also used structural equation modeling (SEM) to derive a total score that weighted some SCI items more than others. For both unit and weighted scoring, a higher score indicated higher well-being.

For the remaining well-being items, responses were assigned values ranging from 1 (for *strongly disagree*) to 7 (for *strongly agree*). Unit scoring was then used to combine responses. Thus, scores could range from 7 to 49 on the SWEMWBS and 8–56 on the FS. For both instruments, a higher score indicated a higher level of well-being. A similar approach was used to score the six SVS items, the four PLQ items, the five GBS items, and the six SJS items. Higher scores for these latter instruments denoted higher levels of energy, direction, belonging, and joy, respectively.

Three trained coders scored the mood descriptions. One score quantified the mood valence as 1 (*negative*), 2 (*negative with reservations*), 3 (*mixed positive and negative*), 4 (*positive with reservations*), or 5 (*positive*). Responses that could not be coded were scored 0. If the coders assigned discrepant values to the same mood description, they discussed their differences to achieve a consensus. Additionally, the coders discussed responses for which they assigned scores of 2 or 4 to collectively decide if those responses should instead receive scores of 1 or 5.

The coders also assigned each mood description a score for each of the four SCI dimensions, as detailed in the preregistration. The scores ranged from 1 (if the response included a negative descriptor for that dimension) to 3 (if the response included a positive descriptor for that dimension). Descriptions were assigned a score of 2 if they contained both positive and negative descriptors. The coders also indicated if the mood description did not mention the SCI dimension in question (scored as 0) or could not be coded (scored as 9). If the coders assigned discrepant scores, they discussed their differences until consensus was achieved.

The coders were blind to the validity testing procedure and worked with a file that contained only the open-ended mood responses, Qualtrics response IDs, and a variable used to sort the data. Thus, their coding decisions should have been unbiased.

### Analyses

All analyses were conducted using Stata 19.0. The output and de-identified raw data are available in the supplemental materials, at https://osf.io/geptf/?view_only=a4a192d940d642278d38cea5fd928132.

Criterion validity was assessed by examining the Pearson correlations between the SCI scores (derived with unit scoring), the valence scores for the mood descriptions, and the SWEMWBS and FS scores. Mood descriptions that could not be coded for valence were excluded from these analyses. Criterion validity was also assessed with a multiple indicators, multiple causes (MIMIC) model. SEM with maximum likelihood estimation was used to determine parameter values. The correlations between the total SCI score and the four underlying dimensions were initially constrained to the same value, which was equivalent to unit-scoring the SCI. The equality constraint was then removed; this was equivalent to weighted-scoring the SCI. The chi-square goodness of fit metrics from the two models were compared to determine how weighted scoring affected the model fit.

Construct validity was assessed by deriving factor scores for the items that assessed each SCI dimension. The Pearson correlation of these factor scores with the corresponding SCI dimension scores was then calculated. Additionally, unequal variances t-tests compared each SCI dimension’s mean score for respondents whose mood description was coded 3 (positive) for that dimension, to the mean SCI score for respondents whose mood description was coded 1 (negative).

## Results

### Criterion validity

All three well-being measures were strongly and approximately equally correlated with mood valence (*r* = .70 for the SCI, *r* = .69 for the SWEMWBS, *r* = .68 for the FS; all *p* < .001). Correlations between SCI unit scores and scores on the two other well-being measures were robust (*r* = .86 for the SWEMWBS, *r* = .88 for the FS; both *p* < .001), while the correlation between the SWEMWBS and FS scores was even higher (*r* = .94, *p* < .001).

**Fig 2:**
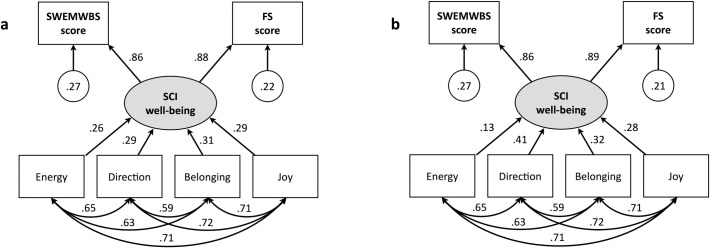
Standardized solution for the MIMIC model with unit scoring (a) and weighted scoring (b). Values in circles are error variances; all other values are correlations.

The standardized solutions for the MIMIC analyses using unit scoring and weighted scoring are shown in Figs 2a and 2b. (The raw score solutions are shown in Fig_S1 in the supplemental materials, at https://osf.io/geptf/?view_only=a4a192d940d642278d38cea5fd928132.) The results mirror those of the correlational analyses. The SCI well-being measure had a correlation of.86 with the SWEMWBS score for both unit and weighted scoring. The correlation with the FS score was.88 with unit scoring and.89 with weighted scoring. The MIMIC model with weighted scoring fit the data better than the model with unit scoring (Δχ^2^ = 90.77, Δ*df* = 2, *p* < .001), implying that the four SCI dimensions contribute differentially to well-being. The correlations of the item responses with the SCI well-being measure ranged from.26 (for energy) to.31 (for belonging) under unit scoring, and from.13 (for energy) to.41 (for direction) under weighted scoring. The correlations between the four SCI dimensions appear at the bottom of Figs 2a and 2b; they ranged from.59 (between direction and belonging) to.72 (between direction and joy).

### Construct validity

There was very strong evidence for the construct validity of the SCI dimensions (Table 2). Correlations between each SCI dimension score and the factor score from other items that assessed the same construct ranged from.80 (for energy) to.85 (for joy). Furthermore, there were large differences in the mean response to each SCI dimension when respondents described their mood as positive for that dimension (mood score = 3) compared to when their mood was negative (mood score = 1). This was reflected in very large effect sizes, ranging from *d* = 2.42 (for direction) to *d* = 3.36 (for joy).

**Table 2:** Correlations of SCI dimensions with construct validity factor scores, and mean SCI item responses for positive and negative mood descriptions.

SCI dimension	Construct validation scale	*r* ^a^	SCI dimension score when mood was negative for dimension			SCI dimension score when mood was positive for dimension			Cohen’s *d*^a^
			*M*	*SD*	*n*	*M*	*SD*	*N*	
Energy	SVS	.80	2.68	0.78	68	4.26	0.50	80	2.47
Direction	PLQ	.81	2.27	0.96	26	4.11	0.71	111	2.42
Belonging	GBS	.84	2.00	0.60	12	4.25	0.70	75	3.28
Joy	SJS	.85	2.02	0.78	51	4.20	0.62	276	3.36

^a^*p* < .001 for all *r* and *d* values.

### Ancillary analyses

Additional analyses beyond those specified in the preregistration were conducted in response to questions from manuscript reviewers. One question was whether our exclusion criteria – which eliminated one third of the respondents who began the survey – may have introduced systematic biases. We could not investigate this using the well-being or mood responses, because of the 282 excluded respondents only 28 completed the entire survey (not counting the 24 duplicate respondents); the remainder skipped some items, abandoned the survey, or were prevented from completing it because they failed an attention check or lived outside the United States. However, most of the excluded respondents provided their gender, level of education, and age before they were excluded, so we examined these three demographic variables.

There were statistically significant differences between the retained and excluded respondents for all three variables (see supplemental materials for details), but this finding should not be characterized as demonstrating bias. To the contrary, applying the exclusion criteria reduced the demographic disparities between the retained respondents and the population they represent: individuals who lived in the United States in 2021 (the year in which the survey was administered). Almost two thirds of the excluded respondents (64.6%) indicated they were male, compared to 55.1% of the retained respondents. Thus, the gender balance was closer in the retained group than in the excluded group to the United States average of 51.0% female in 2021 [41]. Both groups had higher levels of educational attainment than is average in the United States; however, the distribution of the attainment levels was closer to the United States 2021 distribution in the retained group than in the excluded group (Table 3). Finally, respondents in both groups were younger than average for individuals who were living in the United States and aged 18 years or older in 2021 (*M* = 47.96 years [41]), but the retained respondents (*M* = 40.87 years) came closer to this age than the excluded respondents (*M* = 38.31 years).

**Table 3:** Educational attainment levels.

Educational attainment	Excluded respondents (*n* = 237)	Included respondents (*n* = 564)	United States average in 2021 [42]
Did not graduate high school	0.4%	0.9%	9.6%
Graduated high school	13.5%	18.3%	45.4%
Community college degree/certificate	8.0%	17.0%	10.0%
Bachelor’s or similar 4-year degree	54.0%	45.0%	22.2%
Master’s or similar graduate degree	16.9%	15.3%	9.6%
Doctorate or similar graduate degree	6.3%	2.7%	3.2%
Did not respond	0.8%	0.9%	–

The exclusion criteria were not applied to improve the representativeness of the retained sample, even though they had this effect. Instead, they were designed to safeguard the quality of the data by filtering out inattentive respondents, bots, and individuals who misrepresented themselves. To determine how well the exclusion criteria met this goal, we compared the state in which each respondent reported living to the state in which their IP address was located. The outcome for this comparison differed significantly between excluded and retained respondents (see supplemental materials for details). IP addresses could not be determined for 7.0% of the excluded respondents but for only 2.5% of the retained respondents. When the IP address *could* be determined, it matched the reported state far less often for the excluded respondents (36.1%) than for the retained respondents (84.0%). These findings suggest the excluded respondents were much more likely than retained respondents to use virtual private servers to either hide their location or present themselves as living somewhere other than their actual location. These are practices that MTurk workers from overseas locations are suspected of using to access surveys that are restricted to individuals living in the United States [43]. The exclusion criteria appeared to be effective in removing a majority of these questionable respondents.

A second question raised during manuscript review was whether we could provide additional psychometric data to accompany our criterion and construct validity findings. We addressed this by calculating internal reliability metrics for the FS, SWEMWBS, SVS, PLQ, GBS, and SJS in our sample. The values we obtained for Cronbach’s alpha in our sample were high for every scale (Table 4). Furthermore, our values compare favorably to those reported in each scale’s original publication, and to the highest values of Cronbach’s alpha found by ChatGPT 5.0 in a literature search. These findings are especially noteworthy because, unlike most of the other studies that determined alpha values, we intermixed the items from all six scales and presented them in a different, random order to every respondent.

**Table 4:** Values for Cronbach’s alpha.

Scale	Current study	Original study	Highest value
FS	.95	.87 [12]	.95 [44]
SWEMWBS	.91	.84 – 85 [19]	.90 [45]
SVS	.93	.83 –.92 [36]	.92 [36]
PLQ	.92	.82 –.84 [37]	.91 [46]
GBS	.97	.92 –.95 [38]	.95 [38]
SJS	.92	.94 –.95 [39]	.95 [39]

## Discussion

The tests of criterion validity provide strong and consistent evidence that the SCI assesses well-being. The correlations between mood valence and the three well-being measures were all high. Similarly, the correlations between well-being as measured by the SCI and as measured by the SWEMWBS and FS were very high, in both the correlational analysis and the MIMIC analyses.

The correlation between the SWEMWBS and FS scores approached unity, suggesting these two measures are virtually interchangeable. Their intercorrelation was notably higher than their correlations with the SCI score, implying that the SCI assesses well-being somewhat differently. This could be because the SCI is explicitly holistic, whereas the FS and SWEMWBS emphasize EWB. Of the eight items in the FS, only two touch on HWB: The latter portion of “I am a good person and *live a good life*” (emphasis added) echoes Cantril’s Ladder, and “My social relationships are supportive and rewarding” – like the SCI belonging item – taps into both EWB and HWB. Similarly, only one of the seven SWEMWBS item (“I’ve been feeling relaxed”) is arguably more about HWB than EWB, while another (“I’ve been feeling close to other people”) relates to both types of well-being. Future research could provide more insight into the holistic nature of the SCI by cross-validating it with instruments that complement the FS and the SWEMWBS by assessing HWB more strongly than EWB.

Weighted scoring of the MIMIC model found that the SCI direction item correlated with well-being more highly than the other SCI items. However, this does not necessarily imply that direction contributes more to well-being than energy, belonging, or joy; it could be an artifact of the measures chosen to assess the SCI’s criterion validity. The SWEMWBS and FS both contain several items similar to the SCI direction item, which may have increased the importance of direction as estimated by the MIMIC model. Last, there was no substantive difference between unit and weighted scoring of the SCI; both approaches yielded virtually identical correlations with the SWEMWBS and FS scores. Thus, energy, direction, belonging, and joy all contribute to well-being, and unit scoring seems entirely appropriate for the SCI.

The correlations among the four SCI dimensions ranged from moderate to high (.59 to.72), demonstrating that the four dimensions tend to covary. This is not surprising; eudaimonic factors can have hedonic consequences. For example, individuals who lead purposeful lives (EWB) may experience joy (HWB) as a result of this practice. More importantly, the covariance exhibited by the four SCI dimensions suggests that reflective models of well-being are not entirely wrong: Changes in one well-being dimension can be echoed in others. However, the formative model we applied to test the SCI assumes that energy, direction, belonging, and joy *determine* well-being; they are not incidental *consequences* of well-being. This assumption has obvious implications for practitioners who seek to use interventions that target specific elements of well-being.

Accordingly, evidence for the SCI’s construct validity is critical. Correlations of item responses with scales measuring those same dimensions were high. Furthermore, the mean response for each SCI dimension was higher when respondents described their mood positively for that dimension than when they used negative mood descriptions. Collectively, these results demonstrate that the four SCI items do, in fact, assess energy, direction, belonging, and joy.

The formative nature of the SCI and the unique challenges of assessing momentary well-being prevented us from examining several psychometric properties that are typically reported for new instruments. In particular, internal reliability and test-retest reliability were not examined for the SCI because they are not relevant. However, the values we obtained for Cronbach’s alpha in the ancillary analyses were all high and consistent with other published studies using the same instruments, providing general support for the quality of our data.

We excluded one third of the respondents from our analyses. This proportion may seem high, but our exclusion criteria conform with best practices for studies using MTurk samples and our exclusion rate is not unusual [47]. More importantly, the ancillary analyses we conducted suggest the exclusion criteria screened out respondents who may have been misrepresenting themselves. Furthermore, the respondents we retained were more representative of individuals who live in the United States than the respondents we excluded.

Even so, our sample was skewed towards more well-educated respondents; future studies should employ a sample even more representative of the general population. Future research should also track the ethnicity of respondents. Ethnicity data were not collected in this study, so it is not yet known whether individuals from different cultures use the SCI in the same way. Furthermore, repeated administration of some well-being instruments can alter attitudes and lead users to base their ratings on previous responses rather than the anchors specified in the instrument itself [29]. Longitudinal studies are needed to determine if this problem applies to the SCI. This work could also provide evidence that the SCI actually assesses momentary well-being by, for example, demonstrating that stressful events reduce SCI scores while celebratory events increase them.

Tests of the SCI in applied settings are warranted as well. For example, medical patients who are recovering from surgery, therapy clients who are being treated for trauma, and individuals who are undergoing training in highly stressful environments (e.g., military recruits and first responders) could presumably benefit from daily monitoring of their well-being. This would enable interventions or other remedies to be introduced as soon as their need becomes evident. It remains to be seen whether the SCI proves useful in these applications.

In summary, our findings provide strong evidence for the SCI’s criterion and construct validity. These psychometric properties emerge even though the SCI consists of only four items and can be completed in just a few seconds. We therefore recommend the SCI as a tool for momentary well-being assessment and encourage mental health professionals to explore its utility.
